# Phase Ib trial of reformulated niclosamide with abiraterone/prednisone in men with castration-resistant prostate cancer

**DOI:** 10.1038/s41598-021-85969-x

**Published:** 2021-03-18

**Authors:** Mamta Parikh, Chengfei Liu, Chun-Yi Wu, Christopher P. Evans, Marc Dall’Era, Daniel Robles, Primo N. Lara, Neeraj Agarwal, Allen C. Gao, Chong-Xian Pan

**Affiliations:** 1 Division of Hematology Oncology, Department of Internal Medicine, School of Medicine, University of California, Davis, USA; 2 Department of Urologic Surgery, School of Medicine, University of California, Davis, USA; 3 Department of Biochemistry and Molecular Medicine, School of Medicine, University of California, Davis, USA; 4 Office of Clinical Research, UC Davis Comprehensive Cancer Center, Davis, USA; 5 Huntsman Cancer Institute, University of Utah, Salt Lake City, USA; 6 University of California, 4501 X Street, Suite 3016, Sacramento, CA 95817 USA; 7 Present Address: Harvard Medical School, VA Boston Healthcare System, 1400 VFW Parkway, West Roxbury, MA 02132 USA

**Keywords:** Cancer, Prostate cancer

## Abstract

Niclosamide has preclinical activity against a wide range of cancers. In prostate cancer, it inhibits androgen receptor variant 7 and synergizes with abiraterone. The approved niclosamide formulation has poor oral bioavailability. The primary objective of this phase Ib trial was to identify a maximum tolerated dose (MTD) and recommended phase 2 dose (RP2D) of a novel reformulated orally-bioavailable niclosamide/PDMX1001 in combination with abiraterone and prednisone in men with castration-resistant prostate cancer (CRPC). Eligible patients had progressing CRPC, adequate end-organ function, and no prior treatment with abiraterone or ketoconazole. Patients were treated with escalating doses of niclosamide/PDMX1001 and standard doses of abiraterone and prednisone. Peak and trough niclosamide plasma levels were measured. Common Terminology Criteria for Adverse Events (CTCAE) v4.0 and Prostate Cancer Working Group 2 criteria were used to evaluate toxicities and responses. Nine patients with metastatic CRPC were accrued, with no dose-limiting toxicities observed at all dose levels. The recommended Phase II dose of niclosamide/PDMX1001 was 1200 mg orally (PO) three times daily plus abiraterone 1000 mg PO once daily and prednisone 5 mg PO twice daily. Trough and peak niclosamide concentrations exceeded the therapeutic threshold of > 0.2 µM. The combination was well tolerated with most frequent adverse effects of diarrhea. Five out of eight evaluable patients achieved a PSA response; two achieved undetectable PSA and radiographic response. A novel niclosamide/PDMX1001 reformulation achieved targeted plasma levels when combined with abiraterone and prednisone, and was well tolerated. Further study of niclosamide/PDMX1001 with this combination is warranted.

## Introduction

Niclosamide is an oral medication long-approved by the US Food and Drug Administration (FDA) for the treatment of tapeworm infection and is included in the World Health Organization’s list of essential medicines. Numerous studies have since reported that niclosamide is a multi-functional drug with broad anti-tumor activity in almost all major cancer types, including cancer/leukemia stem cells^1,2^. Niclosamide exerts its anti-cancer activity by targeting multiple pathways, including the Wnt/β-catenin, mammalian target of rapamycin complex 1 (mTORC1), signal transducers and activators of transcription 3 (STAT3), nuclear factor-kappa B (NF-κB), Notch pathways and damage to mitochondria^2,3^.

Recently, our group found that niclosamide inhibits prostate cancer growth through targeting androgen receptor (AR) variant 7 (AR-V7)^4–7^. AR comprises three main functional domains: the N-terminal domain (NTD), the DNA binding domain (DBD) and the ligand binding domain (LBD)^8^. Engagement of AR by androgen at the LBD leads to dimerization of AR, nuclear translocation, binding of AR dimers to androgen response elements, and induction of cellular processes. Almost all of the current hormonal therapies for prostate cancer target the AR signaling pathway, including those agents targeting androgen biosynthesis, such as luteinizing hormone-releasing hormone (LHRH) agonists and antagonists, orchiectomy, ketoconazole and abiraterone, and AR antagonists such as bicalutamide, nilutamide, flutamide, enzalutamide, apalutamide and darolutamide. Although many patients initially respond to these therapies, eventually patients develop castration resistant prostate cancer (CRPC). Even with other treatment modalities, including chemotherapy, Radium-223, and sipuleucel-T, the disease remains almost universally lethal^9–11^.

AR-V7 lacks the LBD, is constitutively active and confers resistance of prostate cancer cells to agents targeting the classical AR signaling pathway^12^. Detection of AR-V7 in circulating tumor cells of patients with prostate cancer correlated with resistance to hormonal therapy^13,14^. Through screening of the Prestwick Chemical Library, which contains about 1120 FDA-approved drugs, we found that niclosamide targeted AR-V7^4^, reversed resistance of prostate cancer cells to medications targeting the androgen-AR pathway both in vitro and in vivo, and prolonged the survival of mice carrying resistant prostate cancer xenografts^4–7^.

Even with these strong preclinical data, the clinical application of the existing FDA approved chewable niclosamide tablet has been limited by poor oral availability. To address this absorption issue, we reformulated niclosamide micropowder into capsules and conducted a Phase Ib trial. The objective of this trial is to determine the drug absorption of niclosamide in combination with abiraterone acetate and prednisone in patients with CRPC. Abiraterone in combination with prednisone has been approved by the FDA for the treatment of CRPC and metastatic castration-sensitive prostate cancer^15–17^.

## Materials and methods

This open label, Phase Ib trial began enrollment in October 2016, and currently remains open to accrual for Phase II expansion at the University of California at Davis [clinicaltrials.gov: NCT02807805, registered on June 21, 2016]. This study was approved by the University of California at Davis Institutional Review Board (Protocol No: 871875-2. Initial approval date: March 2, 2016. The protocol is attached as a supplement). Written informed consent was obtained from all enrolled subjects. This clinical trial was performed in accordance with the Declaration of Helsinki. For the Phase Ib portion of this study, patients with progressive mCRPC with a history of cytologically or histologically confirmed prostate cancer were enrolled at University of California Davis. Progressive mCRPC was defined as at least 20% increase of unidimensionally measurable disease despite androgen deprivation therapy (ADT) or at least two consecutive rises in PSA at least 7 days apart while on ADT, within 42 days of enrollment. If PSA was the only indicator of disease progression, PSA was required to be ≥ 5.0.

Patients were required to be ≥ 18 years old, either medically or surgically castrated (with serum testosterone level ≤ 50 ng/dL within 3 months prior to registration), with a Karnofsky performance status ≥ 60%. Patients were required to have adequate organ and bone marrow function (leukocytes ≥ 3000/µL, absolute neutrophil count ≥ 1500/µL, platelets ≥ 100,000/µL, total bilirubin within normal limits, aspartate and alanine transaminase ≤ 1.5 upper limit of normal (ULN), and creatinine ≤ 1.5 ULN). Prior definitive therapies such as radical prostatectomy or radiation therapy were permitted. History of palliative radiation therapy or chemotherapy was also permitted, as long as it was > 4 weeks before the time of enrollment.

Patients with symptomatic disease such that pain was considered uncontrolled were not included; patients with brain metastases were also excluded. Patients were not permitted to have received abiraterone acetate or ketoconazole prior to enrollment to trial; however, other antiandrogen therapy (enzalutamide, nilutamide, flutamide, or bicalutamide) or chemotherapy (docetaxel, cabazitaxel or mitoxantrone) was permitted. A history of HIV or of other malignancies in the past 3 years with the exception of adequately treated cutaneous basal or squamous carcinomas was another exclusion criterium. Patients with known gastrointestinal dysfunction were excluded due to concerns regarding malabsorption.

The dose escalation/de-escalation schedule is shown in Supplemental Information (SI) #1 Table 1. All patients enrolled to the trial were treated with abiraterone acetate 1000 mg by mouth (PO) daily and prednisone 5 mg PO twice a day (bid) starting the same day when niclosamide was started. The study was designed to enroll an initial cohort of 3 patients who were administered the first dose level (DL 0) of niclosamide 400 mg PO bid for 28 days. Niclosamide capsules (PDMX1001) were supplied in 400 mg dosage by PandoMedx. If no Dose Limiting Toxicity (DLT) was observed after at least one cycle of treatment, then intra-patient dose escalation proceeded and the dose of niclosamide would be increased to the next dose level in the same patient until DLT or Dose Level 4 is reached. DLT was defined as any Grade 3, non-hematologic toxicity not reversible to Grade 2 or less within 96 h, or any Grade 4 toxicity. DLT was based on the first cycle of treatment (i.e. one cycle or 4 weeks of treatment) of each dose level. If ≤ 1 DLT were seen in the initial cohort of 3 patients, then 3 additional patients were enrolled. If ≥ 1 DLTs were seen in the initial cohort, then 3 additional patients were enrolled with a starting dose of niclosamide at 400 mg PO bid (DL − 1). Any patients not evaluable for toxicity were replaced. All patients were treated until the development of unacceptable toxicity or until progression of disease.

Pharmacokinetic samples were collected during cycle 2 of niclosamide/PDMX1001 at 1200 mg PO tid, before treatment (trough) and 1 h after niclosamide administration (peak). Ultra high pressure liquid chromatography (UPLC) coupled with MS/MS (mass spectrometry) was performed to determine niclosamide concentration. More detailed information of niclosamide concentration measurements can be found in Supplementary Information #2.

The primary endpoint for the Phase Ib portion of this trial was to establish the maximum tolerated dose (MTD) and recommended Phase 2 dose (RP2D) of niclosamide when combined with abiraterone acetate plus prednisone in patients with mCRPC. Secondary endpoints included pharmacokinetic evaluation of niclosamide, safety as defined by Common Terminology Criteria for Adverse Events (CTCAE) v4.0, and PSA response, defined as a 50% or more reduction from baseline.

## Results

### Patient characteristics

From October 2016 to October 2017, 9 patients were enrolled to the Phase Ib cohort. Median age was 79 (66–88) (Table 1). The majority of patients were Caucasian (n = 6), the remainder were African-American (n = 1), Hispanic (n = 1) and Native-American (n = 1). All patients had metastatic CRPC at the entry of the trial as determined by bone and/or CT scans. All patients received prior ADT ± bicalutamide. Five patients received additional therapies, including enzalutamide/apalutamide (n = 2), docetaxel (n = 3), cabazitaxel (n = 1) and sipuleucel T (n = 1). The treatment cycles ranged from one cycle to over 42 cycles with a median of 6 cycles. Two patients have been on the study for over three years and one patient over 2 years. All three of these patients were still on trial as of December 31. 2019.

**Table 1:** Patient characteristics at the entry of the trial.

Subject no	Age	Racial/ethnicity	Local therapy	Prior treatment	No of cycles	Best response
001	83	Caucasian	Prostatectomy	1998: prostatectomy; 2000: Salvage radiation; since 2004: intermittent ADT; 2015: enzalutamide	1	Unable to determine *Patient received only one cycle with rising PSA
002	66	Hispanic	Definitive radiation	2009: XRT; since 2011: intermittent ADT; 12/2015–09/2016, bicalutamide	42 cycles, ongoing	CR
003	74	Caucasian	Neoadjuvant docetaxel + ADT followed by prostatectomy	2013: neoadjuvant docetaxel + ADT; 2013: RALP; 2014: ADT; 01/15–08/19: bicalutamide	14	PR
004	79	Native American	Prostatectomy	2007: radical prostatectomy; 2013: ADT; 09/2014–04/2016: apalutamide; 07/2016–09/2016: docetaxel	6	SD
005	76	African American	Prostatectomy	2008: prostatectomy; 02/2013: ADT; 03/16–09/19: bicalutamide	32 cycles, ongoing	CR
006	86	Caucasian	None	2015: ADT + bicalutamide	6	PR
007	73	Caucasian	Definitive radiation plus ADT	2015: XRT plus ADT; 1/17: ADT and Deoctaxel, with disease progression; 04/17: cabazitaxel;	2	PD^#^ Possibly neuroendocrine cancer with visceral mets and high chromogranin
008	78	Caucasian	XRT + 6 mo ADT	1998: XRT + 6 mo ADT; 02/2016: ADT; 04/17: Sipeulucel T	3	PD
009	79	Caucasian	None	04/2016: ADT + bicalutamide	18, ongoing	PR

*CR* complete response, *PR* partial response, *SD* stable disease, *PD* progression of disease.

*Patient received only one cycle of treatment at the trial with rising PSA but without clinical progression and was taken off from the trial. Per PCWG2 criteria, if PSA is the only sign of disease progression, patient should be treated for up to 12 weeks.

^#^This patient might have neuroendocrine tumor as he had widespread visceral metastasis and high serum chromogranin.

### Adverse events

Adverse events observed in patients on treatment are summarized in Table 2. No dose-limiting toxicities were observed during the first cycle of treatment at each dose level up to dose level 3 (DL3) with PDMX1001/niclosamide at 1200 mg po tid, abiraterone 1000 mg po qd and prednisone 5 mg po bid. Dose Level 4 (PDMX1001/niclosamide 1600 mg po tid) intra-patient escalation was considered optional as niclosamide levels already reached therapeutic concentration in plasma as shown below. Nevertheless, almost all patients were able to reach DL4. The combination of abiraterone acetate, prednisone, and niclosamide was well-tolerated, with few Grade 3 toxicities. Grade 3 toxicities observed included abdominal pain (n = 1), fatigue (n = 1), hypoalbuminemia (n = 1), anemia (n = 1) and hyperglycemia (n = 1). Of Grade 1–2 adverse events, diarrhea (n = 4), fatigue (n = 3), headache (n = 3), and anorexia (n = 3), were most common. Patient #3 had abdominal discomfort and dizziness which improved after abiraterone was decreased to 750 mg po qd. His PDMX1001/niclosamide dose reached 1600 mg po tid and he tolerated well. No patient discontinued treatment due to toxicity.

**Table 2:** Summary of adverse events.

Adverse event	All grades	Grade ≥ 3
Fatigue	4	1*
Headache	3	
Diarrhea	6	
Dizziness	1	
Dyspepsia	1	
Constipation	2	
Upper respiratory infection	1	
Abdominal pain	1	1*
Nausea	2	
Anorexia	3	
Pruritus	1	
SVT	1	
Mucositis	1	
Edema (limbs)	1	
Fall	1	
Hypoalbuminemia	1	1*
Flatulence	1	
Vomiting	1	
Insomnia	1	
Anemia	1	1*
Hyperglycemia	1	1*

*AE was deemed Grade 3.

### Peak and trough levels of niclosamide

We determined the peak and trough levels of niclosamide in select patients to determine whether the therapeutic concentrations were achieved (Table 3). Plasma samples were collected on 3 patients before (trough) and 1 h after (peak) they took 1200 mg of niclosamide (DL3) after they were treated at DL3 for one cycle. The niclosamide trough concentrations ranged from 0.31 to 0.65 µM (100.1–212.1 ng/mL) while the peak concentrations ranged from 0.21 to 0.72 µM (70.0–236.4 ng/mL). There was no significant difference between the trough and peak niclosamide levels, suggesting that niclosamide reached steady concentrations after one cycle of treatment. Based on our preclinical studies, concentrations of 0.1 µM are sufficient to exert anti-prostate cancer activity, suggesting that this formulation of PDMX1001/niclosamide 1200 mg po tid or higher can potentially be used for future clinical trials.

**Table 3:** PDMX1001/niclosamide trough and peak concentration.

Patient	Level	Time	Concentration (ng/mL)	Concentration (µM)
002	Trough	7.5 h post dosing	100.05	0.306 (trough)
002	Peak	1 h post dosing	70.04	0.214 (peak)
004	Trough	8 h post dosing	162.16	0.496 (trough)
004	Peak	1 h post dosing	170.38	0.521 (peak)
005	Trough	6.5 h post dosing	212.06	0.648 (trough)
005	Peak	1 h post dosing	236.41	0.723 (peak)

### Efficacy

**Figure 1:**
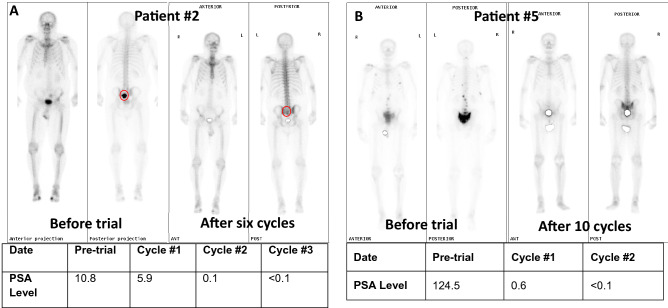
Bone scans and PSA kinetics in two patients with complete PSA response. In both patients, PSA became undetectable after three cycles in the combination treatment. The time of bone scan was indicated in the figure. So far, both patients have been on the study for over 3 years.

Of 9 patients enrolled, 5 patients had ≥ 50% PSA response to treatment, and 2 of these patients had complete PSA response (< 0.01 ng/mL per ultrasensitive PSA test) (SI #3 Table 2). The complete PSA responses were also associated with radiographic improvement per bone and CT scan (Fig. 1). These two patients have been on the protocol treatment for over three years and are still on the trial as of December 31, 2019. One of the patients with a complete PSA response is patient #2 whose niclosamide trough and peak levels were 0.31 and 0.21 µM, respectively. This is consistent with our preclinical studies suggesting that niclosamide at the level of 0.1 µM is sufficient to inhibit AR-V7.

Of the three patients who did not achieve a PSA response, patient #4 did not meet criteria for PSA response, yet the only radiographically apparent metastatic lesion was biopsied and confirmed to be completely necrotic without any viable cancer cells. There was suspicion that Patient #7 might have had neuroendocrine differentiated CRPC, as he was noted to have numerous, large, necrotic hepatic metastases in addition to many other large necrotic lymph nodes throughout chest and retroperitoneum on CT imaging, while PSA was low (31 ng/mL) and chromogranin was high.

Patient #1 was taken off from the trial after only one cycle of treatment with rising PSA due to patient preference without any clinical progression and was not included in response analyses. Hence he was considered non-evaluable by Prostate Cancer Working Group 2 (PCWG2) criteria, which specify that patients could have rising PSA during the first 12 weeks of treatment and should remain on treatment unless they experience clinical progression.

## Discussion

The results from the Phase Ib portion of this ongoing study demonstrate that abiraterone acetate, prednisone, and niclosamide can be safely combined in patients with mCRPC. Our findings suggest that at DL 3 with niclosamide 1200 mg PO tid, and the standard dose of abiraterone 1000 mg PO daily, and prednisone 5 mg PO bid, few Grade 3 toxicities were observed. In addition, at DL3, plasma niclosamide levels reached therapeutic concentrations based on preclinical studies and thus was deemed the recommended Phase 2 dose (RP2D), though many patients could safely be treated at DL 4. In previous preclinical studies, the anti-tumor effects of niclosamide in abiraterone-resistant models were observed as low as 0.1 µM^5^, and were consistently noted when the concentration reached 0.25 µM^4–7^. In this study, concentrations were achieved in the therapeutic range at RP2D, and thus the Phase II portion of the study is continuing to enroll at that dose level.

Even though this Phase Ib trials focused on CRPC, its impacts could be extended to other cancers as an oncology therapeutic. As discussed above, niclosamide has been shown to be effective in targeting multiple pathways across almost all major cancers that have been tested so far^1,2^. However, because of the perception that niclosamide is not orally absorbable, only one clinical trial in cancer patients has been reported and another has been designed^18,19^. Here we have shown that both the trough and peak levels of PDMX1001/niclosamide at the well-tolerated dose of 1,200 mg PO tid exceed the effective dose level of 0.1 µM and (Table 2), in most cases (except one peak level), 0.25 µM, a level with consistent in vivo preclinical anti-tumor activity^4–7^. At this dose level, no DLTs were observed. In fact, two patients (patient #2 and 5) were treated with PDMX1001/niclosamide 1600 mg PO tid for over two years without significant toxicity.

In this study, we did not observe any difference between peak (1 h after oral administration) and trough (just before dosing) drug concentration after 1 cycle of treatment with PDMX1001/niclosamide PO tid. We hypothesize that this stable drug concentration is secondary to continuous absorption after dissolution of the PDMX1001/niclosamide capsule, which releases niclosamide into the gut.

The combination of reformulated PDMX1001/niclosamide, abiraterone and prednisone is well tolerated. Our findings are in contrast to a recent study which evaluated the combination of enzalutamide and commercially available niclosamide in patients with mCRPC^18^. In that study, two patients were treated with standard-dose enzalutamide (160 mg PO once daily) and niclosamide 1000 mg PO tid, with 2 DLTs observed: Grade 3 nausea, vomiting and diarrhea in one patient, and Grade 3 colitis, abdominal pain and diarrhea in the other patient. Hence, niclosamide 500 mg PO tid daily was considered to be the RP2D which is much lower than the RP2D of our study at 1200 mg PO tid. At this time, it is not clear whether the sizable difference of tolerable niclosamide dose between these two trials is secondary to the choice of combination therapy (enzalutamide versus abiraterone plus prednisone) or differences in formulation.

Even though a Phase Ib trial is not designed to establish efficacy, our study suggests that the combination of PDMX1001/niclosamide, abiraterone and prednisone is clinically active in CRPC. Of the nine patients enrolled in this Phase Ib trial, two out of nine patients achieved complete PSA response (< 0.2 ng/mL, Supplementary Information #3 Table 2) associated with dramatic radiographic response (Fig. 1). In fact, both patients achieved completely undetectable PSA (< 0.01 ng/mL) when they were tested with ultrasensitive PSA testing four cycles after enrollment into the trial. These patients have also enjoyed sustained responses and remain on treatment > 3 years after enrollment.

One limitation of this study is its small size. Thus conclusions cannot be reached regarding the efficacy beyond the clinical activity of the combination. This study also utilized intrapatient dose escalation, which can expose a greater proportion of patients to higher doses of drug and has the advantage of allowing data from all patients to be utilized in determining the RP2D. However, this approach can theoretically mask cumulative or delayed toxicities at a dose level, and may limit interpretation of the results as compared to standard 3 + 3 design. Nevertheless, our data do indicate that reformulated PDMX1001/niclosamide can be absorbed after oral administration to reach therapeutic concentration which supports further study of niclosamide in the treatment of other cancers.

In conclusion, the combination of PDMX1001/niclosamide, abiraterone and prednisone is well tolerated. At the RP2D of PDMX1001/niclosamide at 1200 mg PO tid, therapeutic concentration of niclosamide can be achieved. Further clinical trials are warranted and are currently ongoing. Reformulated PDMX1001/niclosamide is a potential candidate to be tested in other cancer types with its promising pharmacokinetics.

## Supplementary Information

Supplementary Information.
