# Supplementary material for: Phase Ib trial of reformulated niclosamide with abiraterone/prednisone in men with castration-resistant prostate cancer
Source: Sci Rep. 2021 Mar 18;11:6377. doi: 10.1038/s41598-021-85969-x (PMC7973745; doi:10.1038/s41598-021-85969-x)
Supplement: Supplementary file 1 — Supplementary Information. [file 41598_2021_85969_MOESM1_ESM.docx]

**Supplemental Information (SI)**

**Phase Ib trial of reformulated niclosamide with abiraterone/prednisone in men with castration-resistant prostate cancer**

Mamta Parikh, MD,MS*

School of Medicine

Department of Internal Medicine, Division of Hematology Oncology

University of California, Davis

Chengfei Liu, MD, PhD*

School of Medicine

Department of Urologic Surgery

University of California, Davis

Chun-Yi Wu, PhD

School of Medicine

Department of Biochemistry and Molecular Medicine

University of California, Davis

Christopher P. Evans, MD

School of Medicine

Department of Urologic Surgery

University of California, Davis

Marc Dall’Era, MD

School of Medicine

Department of Urologic Surgery

University of California, Davis

Daniel Robles

Office of Clinical Research

UC Davis Comprehensive Cancer Center

Primo N. Lara, MD

School of Medicine

Department of Internal Medicine, Division of Hematology Oncology

University of California, Davis

Neeraj Agarwal, MD

Huntsman Cancer Institute

University of Utah

Allen C. Gao, MD, PhD

School of Medicine

Department of Urologic Surgery

University of California, Davis

Chong-Xian Pan, MD, PhD

School of Medicine

Department of Internal Medicine, Division of Hematology Oncology

University of California, Davis

**SI #1. Table 1. Dose escalation schedule**

| **Supplemental Information #2 Table 1. Dose Escalation Schedule** | | | |
| --- | --- | --- | --- |
| **Dose Level** | **Dose** | | |
|  | ***PDMX1001/niclosamide^&^*** | ***Abiraterone*** | ***Prednisone*** |
| Level -1 | 400 mg po qd | 750 mg po qd | 5 mg po bid |
| Level 0 | 400 mg po bid | 1,000 mg po qd | 5 mg po bid |
| Level 1 | 800 mg, po bid | 1,000 mg po qd | 5 mg po bid |
| Level 2 | 800 mg, po tid | 1,000 mg po qd | 5 mg po bid |
| Level 3 | 1,200 mg, po tid | 1,000 mg po qd | 5 mg po bid |
| Level 4 | 1,600 mg, po tid | 1,000 mg po qd | 5 mg po bid |
| &: PDMX1001/niclosamide: Patients was started on PDMX1001/niclosamide at 400 mg orally twice daily. If no grade 3 or higher toxicity associated with PDMX1001/niclosamide is seen for at least one cycle (4 weeks) at each dose level, patients can be advanced to the next dose level . | | | |

**SI #2. Detect of niclosamide concentration by UPLC coupled with MS/MS**

Pharmacokinetic samples were collected during cycle 2, before treatment (trough) and one hour after niclosamide administration (peak). To quantify niclosamide concentration in clinical samples, human plasma calibrators at the following niclosamide concentrations were made for establishing a human plasma calibration curve: 0, 0.5, 1, 5, 10, 50, 100, 500, and 1000 ng/ml. All were prepared in blank human plasma with lithium heparin as the anticoagulant (Bioreclamation IVT, Westbury, NY). The resulting human plasma standard calibrators and clinical trial patient plasma obtained from the hospital were loaded into each well of a 96-well Isolute PLD+ protein and phospholipid removal plate (Biotage, Uppsala, Sweden). The internal standard (IS), 13C6-niclosamide (Millipore Sigma, St. Louis, MO), 100 ng/ml, was then added to each well. A house vacuum was then applied to draw the mixture from each well passing through the built-in filter and the resulting filtrate directly dripped into each well of the filtrate collecting plate underneath. Five microliter of the resulting filtrate was injected into a Waters (Milford, MA) Acquity UPLC with a BEH C18 1.7 µm, 2.1 mm × 50 mm column. The flow rate was 0.5 ml/min and the column temperature was set at 40 °C. The autosampler temperature was set at 10 °C. The following LC gradient program was used for the separation: 0-1.5 min, 10%B; 1.51-3.5 min, 95%B; 3.51-5 min, 10%B. The output of the UPLC was fed to a Waters Xevo TQ-S triple quadrupole MS/MS system, which was used to ionize target molecules with the ESI- probe and monitor the ion m/z fragmentation transitions from 325 → 171 for niclosamide quantification, and 331 → 177 for 13C6-niclosamide quantification at multiple reaction monitoring (MRM) mode. The calibration curve was fitted with weighted (1/x^2^) least-squares linear regression algorithm. The extraction yield was 58.29 ± 9.45% and the matrix effect enhanced the niclosamide MS signal by 25.16%. Both inter- and intra-batch accuracy were lower than 10% (%deviation) and both intra- and inter-batch precision were also lower than 10% (%CV). The inter- and intra-batch accuracy and precision for the LLOQ (0.5 ng/mL) are all between 10~11%.

**Supplement #3**

| **Supplemental Information #3 Table 2. Comparison of PSA response between abiraterone plus PDMX1001 and historical control** | | | | |
| --- | --- | --- | --- | --- |
| **Treatment** | **Non-response**  (PSA increase or PSA decrease of <50%) | **Response** | | |
|  |  | **Partial response**  (PSA decrease >=50% but >0.2) | **Complete response**  (PSA≤0.2) |  |
| **Abiraterone alone**  **(historical control)*** | 15 | 15 | 0 |  |
| **Abiraterone plus PDMX1001^#^** | 3 | 3 | 2^&^ |  |
| *: These 30 CRPC patients were treated with abiraterone plus prednisone at University of California Davis during 2018. There were used as historical controls.  ^#^: Of the 9 patients enrolled int his trial, 8 were included for the response analysis. Patient #1 was prematurely taken off from the trial after only one cycle of treatment with rising PSA without any clinical progression, and was not included in the response analyses.  ^&^: In both cases, PSA was less than 0.01 as determined by ultrasensitive PSA tests. | | | | |
